# Carriage rate and serotypes of *Streptococcus pneumoniae* amongst children in Thika Hospital, Kenya

**DOI:** 10.4102/ajlm.v2i1.45

**Published:** 2013-05-20

**Authors:** Susan Githii, Gunturu Revathi, Anne Muigai, Samuel Kariuki

**Affiliations:** 1 Jomo Kenyatta University of Agriculture and Technology, Itromid KEMRI, Kenya; 2 The Aga Khan University Hospital, Nairobi, Kenya; 3 Jomo Kenyatta University of Agriculture and Technology, Juja, Kenya; 4 Centre for Microbiology Research, KEMRI, Kenya

## Abstract

*Streptococcus pneumoniae* is a major cause of morbidity and mortality worldwide. Rates of carriage are highest in infants and the elderly. The objectives of this study were to determine the rate of nasopharyngeal colonization by *S. pneumoniae*, and to describe the antibiotic resistant patterns and the serotypes of the carried isolates. A cross-sectional study design was used. Nasopharyngeal swabs were collected from 315 children in the months of October and November 2010 and processed to isolate *S. pneumoniae*. The isolates were serotyped by the Quellung reaction and their antibiotic susceptibilities assessed by the disc diffusion method. The overall nasopharyngeal carriage rate for *S. pneumoniae* was 17%. Seventeen serotypes were detected amongst 55 strains analysed: 6A, 23F, 19F, 13, 6B, 14A, 20, 7C, 1, 15B, 35B, 19A, 11A, 34, 5, 3 and 23A. Susceptibility testing revealed that nearly all (98%) were resistant to cotrimoxazole, 9% were resistant to penicillin and 7% to cefotaxime. Resistance to chloramphenicol and erythromycin was 2% and 4%, respectively. All isolates were fully sensitive to tetracycline.

High levels of cotrimoxazole resistance and some resistance to other antimicrobial agents commonly used in Thika District Hospital shows that there is need to revise antimicrobial policy in this region in the treatment of invasive pneumococcal infections. The frequent serotypes found in this study have previously been associated with pneumococcal infections in children. Several of these serotypes are included in the ten-valent vaccine and therefore use of this vaccine will help reduce pneumococcal infections in Thika.

## Introduction

*Streptococcus pneumoniae* is an inhabitant of the human upper respiratory tract.^1^ The main reservoir of *S. pneumoniae* is the human nasopharynx and this is the main source of person to person transmission.^1^ Pneumococci are usually spread through respiratory secretions and aerosols and give rise to an invasive infection only after spreading to other areas of the respiratory tract, or, if they penetrate the nasopharyngeal mucosa, leading to systemic circulation *via* the cervical lymphatic.^2^ Carriage rates differ depending on environment and age, with the highest carriage rates occurring in children between two and four years old.^3^ The risk of disease is highest amongst children below five years old, older adults, smokers, and persons with certain chronic illnesses.^4^ Children under five years old are frequently colonised for prolonged periods with the same serotypes.^5^ Only a small percentage of colonised children develop an invasive infection, but pneumococcal nasopharyngeal isolates reflect the strains currently circulating in the community.^1^ Treatment of pneumococcal infections is dependent on the site of infection and currently involves administration of antimicrobial agents and immunisations with the pneumococcal polysaccharides vaccines.^6^ The most common agents currently being used by physicians to treat patients are the beta-lactam antibiotics which include penicillins, cephalosporins, monobactams and carbapenems.^7^ Other antibiotics include the macrolides and fluoroquinolones.^8^

*S. pneumoniae* has demonstrated increasing resistance to the commonly used antibiotics, especially penicillin.^9^ These antibiotic-resistant strains are more frequently found in child carriers than in adult carriers.^10^ Antibiotic-resistant *S. pneumoniae* strains are usually associated with a limited number of serotypes which are frequent causes of invasive paediatric infections.^11^ Thus, an understanding of nasopharyngeal carriage prevalence, serotype distribution and antibiotic susceptibility patterns to commonly used antimicrobial agents in various communities is of great importance.

## Ethical considerations

Ethical clearance for the study was obtained from Kenya Medical Research Institute (KEMRI) Scientific Steering Committee and Ethical Review Committee. Approval was also obtained from Medical superintendent, Thika District Hospital.

## Methods

### Study site

The study was conducted in Thika District Hospital in Thika, Kenya, during the months of October and November 2010.

### Study population

The study population included children of five years and below who had been admitted to Thika District Hospital, pediatric ward and whose parent or guardian had given consent for participation in the study.

### Study design

A cross-sectional study design was used.

### Study sample size

The sample size calculation was based on the precision around the estimate of the expected (28%) nasopharyngeal carriage rate of S. *pneumoniae*, using the formula *N* = Z^2^ *P* (1-*P*)/0.05.^12^ This resulted in a minimum sample size of 310 children.

### Study information and consent from parents or guardians

Selection of the patients was done from the hospital’s database and it involved those children who had been admitted to the hospital on the previous day. The parents or guardians of the selected children were called to the procedure room and information sheets explaining the purpose of the study and the procedures involved were distributed. Those who allowed their children to participate in the study were given a consent form to sign.

### Obtaining nasopharyngeal swabs

Nasopharyngeal samples were collected by gentle insertion of a flexible swab stick with Dacron-tipped flexible-wire swabs. This was done by tipping the child’s head slightly backwards, and passing the swab directly backward, parallel to the floor of the nasopharynx. The swab was passed till it reached the posterior pharynx. Once in place, the swab was rotated through 180 degrees and left in place for two seconds to saturate the tip before removing it slowly. Once the nasopharyngeal specimen was collected, it was placed in a bottle containing 3.0 mL of Trypticase soy broth medium and labelled with a study specimen number. Excess wire handle was cut off from the swab leaving the swab itself in the transport media and the cap was tightened.

## Laboratory procedures

### Processing of nasopharyngeal swabs

The specimen was vortexed for 20 seconds to disperse organisms from the swab stick. Ten microlitres of the specimen was inoculated on Columbia blood agar plates (BA) and incubated at 35 °C in 10% carbon dioxide overnight. The plates were checked for growth at 24 and 48 hours. The identity of the isolates was confirmed by standard laboratory methods, which included colony morphology, gram staining, catalase tests, susceptibility to ethylhydrocupreine hydrochloride (optochin) and bile solubility tests.

### Preservation of *Streptococcus pneumoniae* strains

Pneumococcal isolates were stored by picking from the optochin plate one or more colonies and streaking them as a lawn onto a BA plate. After overnight incubation, the growth was examined for purity. Pure growth was harvested with a sterile swab and dispensed into tubes of Trypticase soy broth medium. The suspension was then stored at −20 °C and −70 °C for later serotyping.

### Antimicrobial susceptibility testing

Antimicrobial susceptibility testing was determined using Kirby-Bauer disc diffusion technique for the confirmed *S. pneumoniae* isolates. A small inoculum of each bacterial isolate was emulsified in 3 mL sterile normal saline in Bijou bottles and the density was compared with a barium chloride standard (0.5 McFarland). A sterile cotton swab was dipped into the standardised solution and used for evenly inoculating Mueller–Hinton plates. The plates were then allowed to dry. Antibiotics with the following concentrations were placed on the plates: chloramphenical 30 µg, cotrimoxazole (sulfamethoxazole 23.7 µg, trimethoprime 1.25 µg), tetracycline 30 µg, cefotaxime 30 µg, erythromycin 15 µg and penicillin 10 IU. The antibiotics were well spaced in order to prevent the overlapping of inhibition zones. These antibiotics were selected based on the prescription practices in Thika District Hospital. The plates were incubated at 35 °C for 24 hours. Quality control for susceptibility patterns was performed on a daily basis using *S. pneumoniae* ATCC 49619. Susceptibility results were interpreted according to the Clinical Laboratory Standard Institute Guidelines, 2008.

### Serotyping of pneumococci by latex agglutination method

Pneumococci were serotyped by the latex agglutination method using commercially available antisera from Staten’s Serum Institute, Copenhagen, Denmark. The serotypes were defined by the Quellung reaction. A loopful of overnight growth of pure *S. pneumoniae* was taken from a BA plate and suspended in 300 μL of phosphate buffered saline, pH 7.2. On a glass slide, 5 μL of latex reagents and 10 μL of cell suspension were mixed with a pipette tip and the slide rocked for one minute. The slide was observed for any sign of visible clumping of the suspension. Clumping of the cells indicated a positive result implying presence of the pneumococci; smooth suspension indicated negative results, implying absence of the pneumococci. This procedure was repeated until the specific pool was known. Once the pool was known, each type or group within the pool was tested individually. The types that historically occurred more often were tested first followed by the next ones in the order of their occurrence. All the pneumococcal strains were then identified finally by the Quellung test.

### Quellung reaction for identification of pneumococcal factors

The cell suspension prepared above was mixed with the respective factor sera to determine its factors. Four microlitres of bacterial cell suspension was dispensed onto a labelled slide. Three microlitres of the antiserum were added and mixed thoroughly with the cell suspension. A cover slip was placed on the mixture whilst avoiding drying. Using an oil immersion lens, the mixture was examined under a phase contrast microscope for the presence of apparent capsular swelling to identify the pneumococcal factors.

### Statistical analysis

Data entry and analysis were performed using Microsoft Excel.

## Results

### *Streptococcus pneumoniae* carriage rate

Nasopharyngeal swabs were collected from 315 children: 185 males and 130 females. *S. pneumoniae* was isolated from the nasopharynx in 55 of the 315 children, representing a carriage rate of 17%. There was no significant difference in carriage rates between males and females. The highest rate of carriage was found in children between one and two years old.

Antibiotic susceptibility testing was performed on all of the 55 isolates. The proportion of isolates classified as susceptible, intermediate or resistant to the antibiotics that were tested was determined (Table 1).

**TABLE 1:** Anti-microbial susceptibility of *Streptococcus pneumoniae* isolates.

Drugs	Resistant		Intermediate		Sensitive	
	Frequency	Percentage (%)	Frequency	Percentage (%)	Frequency	Percentage (%)
Penicillin	5	9	0	0	50	91
Tetracycline	0	0	0	0	55	100
Cefotaxime	4	7	0	0	51	93
Erythromycin	2	4	0	0	53	96
Chloramphenicol	1	2	0	0	54	98
Cotrimoxazole	54	98	0	0	1	2

### Serotype distribution

Serotyping was performed on all of the 55 isolates. A total of 17 serotypes were identified in this study: Nine (16%) serotype 6A, eight (15%) serotype 23F, seven (13%) serotype 19F, five (9%) serotype 13, four (7%) serotypes 6B and 14A, two (4%) each of serotypes 20, 7C, 1, 15B, 35B, 19A and 11A, and one (2%) each of serotypes 34, 5, 3 and 23A (Table 2).

**TABLE 2:** Distribution of the identified *Streptococcus pneumoniae* serotypes.

Serotype	Frequency	Percentage distribution (%)
6A	9	16
23F	8	15
19F	7	13
13	5	9
6B	4	7
14A	4	7
20	2	4
7C	2	4
1	2	4
15B	2	4
35B	2	4
19A	2	4
11A	2	4
34	1	2
5	1	2
3	1	2
23A	1	2

**Total**	**55**	**-**

## Discussion

### Pneumococcal carriage rate

The 17% prevalence of nasopharyngeal colonisation by *S. pneumoniae* observed in this study was substantially lower than previous studies. The study was conducted in the months of October and November which was the rainy season. In a study conducted in Kilifi, Kenya, a carriage rate of 53% and 62% during the dry and rainy season was observed respectively,^13^ which was much higher than the findings in this study. A 36% carriage rate was observed in children attending the Maternal Child Health Clinic (MCH) in Eldoret, Kenya, which was also much higher than the findings in this study.^14^ Colonisation prevalence varies across different regions, depending upon climatic and geographic location.^3^ This fact may partially explain the differences in the carriage rates.

### Antimicrobial susceptibility to penicillin

In the present study, 9% of *S. pneumoniae* cases were resistant to penicillin. This is much lower than 25% resistance observed in a study amongst adult patients attending clinics in Nairobi, Kenya,^15^ but similar to the 10% resistance reported in Canada.^16^ Resistance to penicillin occurs when the target penicillin binding proteins become physically altered so that penicillin binds with reduced affinity.^17^ Penicillin has been the drug of choice for pneumococcal infections because of its efficacy, safety and low cost; thus significant levels of resistance would have substantial implications for pneumonia treatment.

### Chloramphenicol

Resistance to chloramphenicol was 2%. This correlates with findings in rural western Kenya in which resistance to chloramphenicol was also found to be 2%.^18^ Low resistance to chloramphenicol (4%) had also been found in the Eldoret, Kenya, study.^14^ Chloramphenicol therefore still finds a role in controlling pneumococcal respiratory infection. Resistance to chloramphenicol in the pneumococcus is mediated by the acquisition of a gene encoding chloramphenical acetyl transferase. This gene is located on a plasmid, probably derived from the Staphylococcus, which has entered the pneumococcus by linearisation and inclusion on a transposable element.^19^

### Cotrimoxazole

A high rate of cotrimoxazole resistance (98%) was observed amongst the pneumococcal isolates. A study on pneumococcal bacteremia conducted in rural western Kenya also had observed similary high resistance of 95%. Cotrimoxazole is amongst the most commonly available antibiotic in most public hospitals for treatment of acute respiratory tract infections and other bacterial infections, and the high level of resistance may reflect overuse and sometimes its misuse.

Also, in many developing countries, antimicrobials are purchased in single doses and taken only until the patient feels better, which may occur before the pathogen has been eliminated.

### Erythromycin

The study revealed 2 cases resistant to erythromycin. A study carried out on adult patients attending clinics in Nairobi, Kenya, did not find any erythromycin resistance.^15^ Resistance to erythromycin varies worldwide with low resistant rates being observed. Resistance to erythromycin can result from mutation in chromosomal genes or plasmids transferred from resistance bacteria. The low resistant rate observed in this study suggests that erythromycin can continue to be used as empiric therapy for pneumococcal infections.

### Cefotaxime

The 7% resistance to cefotaxime found in this study is higher than in a study conducted in Italy.^21^ Cefotaxime is used as an optional alternative to penicillin-resistant strains and also to those individuals who are allergic to penicillin. The resistance noted to cefotaxime in this study was low, perhaps because the drug is expensive and therefore less abused in this region.

### Tetracycline

A study carried out in Kenya amongst adults with acute pneumonia had observed resistance of 32%.^22^ As the isolates in this study showed no resistance to tetracycline, this antimicrobial would be useful for treatment of pneumococcal infections, but as there have been reported cases of resistance elsewhere, regular monitoring of resistance to tetracycline will be necessary in Thika.

### Prevalent serotypes

Serotypes 1, 5, 6A, 6B, 14A, 19F and 23F identified in this study account for the majority of invasive infections in most regions and are commonly involved in invasive pneumococcal diseases.^23^ In a study conducted in Kilifi, Kenya, serotypes 19F, 6B, 23F and 6A were found to be the most prevalent, which is comparable to the findings in this study.^13^ Serotype 19F was found in 13% of the samples. This serotype is more frequently found amongst children.^24^ The serotypes 19F, 23F, 6B, 1 and 5 identified in this study are included in the ten-valent vaccine and therefore they can be prevented by vaccination. Serotype 6A was the most prevalent in this study but is not included in the ten-valent vaccine currently used in Kenya. This warrants evaluation of alternative vaccines that may protect against a wider range of serotypes.

## Conclusion

Antimicrobial resistance observed in this study shows that there is need to revise antimicrobial policy in this region in the treatment of invasive pneumococcal infections. Surveillance programmes should be carried out continuously to monitor the susceptibility levels of *S. pneumoniae* strains in this region in order to curb the problem of developing resistance at an early stage. Some of the serotypes identified in this study are included in the ten-valent vaccine which is currently being used in Kenya. Therefore use of this vaccine will help in the prevention and reduction of pneumococcal infections in Thika. Vaccines that protect against other common serotypes would also be beneficial.
